# An Inherited Small Microdeletion at 15q13.3 in a Patient with Early- Onset Obsessive-Compulsive Disorder

**DOI:** 10.1371/journal.pone.0110198

**Published:** 2014-10-10

**Authors:** Carolina Cappi, Ana Gabriela Hounie, Daniel B. Mariani, Juliana Belo Diniz, Aderbal R. T. Silva, Viviane N. S. Reis, Ariane F. Busso, Amanda Gonçalves Silva, Felipe Fidalgo, Silvia Regina Rogatto, Euripedes C. Miguel, Ana C. Krepischi, Helena Brentani

**Affiliations:** 1 Institute and Department of Psychiatry, São Paulo University Medical School, São Paulo, Brazil; 2 Inter-institutional Grad Program on Bioinformatics, Institute of Mathematics and Statistics, São Paulo University, São Paulo, Brazil; 3 International Research Center, AC Camargo Cancer Center, São Paulo, Brazil; 4 School of Medicine, São Paulo State University, Botucatu, São Paulo, Brazil; 5 Federal University of São Paulo-UPIA-UNIFESP, São Paulo, Brazil; University of Illinois at Chicago, United States of America

## Abstract

Copy number variations (CNVs) have been previously associated with several different neurodevelopmental psychiatric disorders, such as autism, schizophrenia, and attention deficit hyperactivity disorder (ADHD). The present study consisted of a pilot genome-wide screen for CNVs in a cohort of 16 patients with early-onset obsessive-compulsive disorder (OCD) and 12 mentally healthy individuals, using array-based comparative genomic hybridization (aCGH) on 44K arrays. A small rare paternal inherited microdeletion (∼64 kb) was identified in chromosome 15q13.3 of one male patient with very early onset OCD. The father did not have OCD. The deletion encompassed part of the *FMN1* gene, which is involved with the glutamatergic system. This finding supports the hypothesis of a complex network of several genes expressed in the brain contributing for the genetic risk of OCD, and also supports the glutamatergic involvement in OCD, which has been previously reported in the literature.

## Introduction

Twin and family studies provide strong evidence of the importance of genetic factors for the expression of obsessive-compulsive disorder (OCD) and other disorders in the obsessive-compulsive spectrum [1]. To date, more than 100 positional and functional candidate genes related to serotonin, dopamine, and glutamate transmission have been implicated in these disorders [2]. However, most of the findings have not been replicated. A recent meta-analysis of all genetic association studies of OCD, which provided sufficient information for data extraction, found that the disorder was associated with serotonin-related polymorphisms (*5-HTTLPR* and *HTR2A*) and that there may be significant sex differences in the genetic bases of the disease, with only male OCD patients carrying polymorphisms involved in catecholamine modulation (*COMT* and *MAOA*) [3]. Secondary analyses of polymorphisms examined in fewer than five data sets identified other candidate polymorphisms associated with trophic factors, neurotransmitters, and with an immunologic factor [3].

The first Genome-Wide Association Study (GWAS) of OCD, which was recently published, analyzed 400 trios, 1,465 cases and 5,557 controls having found no associations between any SNPs (single nucleotide polymorphisms) and OCD at a genome-wide significant level in the combined trio-case-control sample. It is worth mentioning, however, that the top-ranked SNPs were related to transcriptional regulation and to glutamatergic neurotransmission and signaling [4].

Recently, the original genetic model for psychiatric disorders, which explained these conditions as resulting from the effect of common genetic variants (>1% frequency), has been challenged by a model involving high impact variants which are individually rare, but collectively common [5]. Research has shown that, although SNPs outnumber CNVs (copy number variations) in the genome by three orders of magnitude, their relative contribution to genome variation is similar [6]. Therefore, as could be expected from these observations, the study of CNV has allowed a more comprehensive understanding of disease etiology, bringing rare variants to the forefront [7].

Early studies analyzed CNVs ranging from one kilobase (kb) to several megabases (Mb) in size, including large insertions or deletions in the genome. However, the increased accuracy of detection techniques has allowed the identification of smaller CNVs, so that many recent studies involve the analysis of fragments smaller than 1 kb [8].

CNVs are especially relevant to the study of mental disorders, as they have been causally associated with neurodevelopmental disorders and learning disabilities [9]. Specific rare CNVs have also been found to be associated with autism, schizophrenia, and attention-attention deficit hyperactivity disorder (ADHD) [10]–[14].

The current hypothesis for the etiology of mental disorders suggests that these conditions are polygenic, and that both common and rare variations contribute to their development. Therefore, rather than consisting of risk factors for specific conditions, CNVs are thought to confer broad susceptibility to a variety of neurodevelopmental disorders. Large, rare CNVs are more pathogenic, and are more likely to be under negative evolutionary selection [15]. Although CNVs with smaller effect sizes may also have an important role in the etiology of mental disorders, their individual influences and the impact of their interaction with other CNVs on the development of mental illnesses have been poorly studied [16].

To date, few studies have investigated CNVs in OCD patients. One study was conducted to search for CNVs in these individuals screened for 15q11-13 and 22q11.2 microrearrangements associated with Prader-Willi and DiGeorge Syndromes (DGS). The study was performed in 236 OCD probands using Multiplex Ligation-dependent Probe Amplification (MLPA), which allows the relative quantification of the copy number in at least 50 sequences in a single experiment. The study did not detect any CNVs in these regions, possibly due to the rare prevalence of these chromosomal anomalies in OCD and due to the exclusion of significant mental retardation in the samples studied [17]. Studies have shown that pathogenic CNVs are more prevalent among the individuals with moderate and severe intellectual disability, which are usually exclusionary criteria of research protocols [18].

Another study by Hooper et al., (2012) used cytogenetic analysis and fluorescence in situ hybridization (FISH) to identify an apparently balanced cytogenetic chromosome translocation t(6;22)(q16.2;p13) that segregated from the mother with OCD to her male son with Tourette Syndrome (TS) and OCD. Whole genome mate-pair sequencing and high resolution SNP array analysis was performed in the male proband to identify any related or additional sub-microscopic rearrangements. The largest heterozygous deletion identified in this secondary analysis was a 400 kb deletion located 1.3 Mb telomeric to the chromosome 6q breakpoint. This deletion caused heterozygous loss of all coding sequences of the *GPR63*, *NDUFA4* and *KLHL32* genes which are expressed in the brain [19]. In addition, this deletion coincides with a rearrangement previously reported in a girl diagnosed with Autism Spectrum Disorder (ASD) and developmental delay [20].

In a study of 136 cases of children and adolescents with OCD compared with 106 healthy age-matched controls, Walitza et al., (2011) reported that the deletion (one copy) of a small (100 bp) CNV localized near (68-bp upstream the rs 6311 in the promoter region of the *HTR2A* gene was associated to a very early-onset and to symptom severity of OCD. The frequency of this deletion was increased in the OCD group (n = 8) when compared to healthy controls (n = 1), and carriers of one copy (deletion) of the CNV were associated with a very-early-onset OCD and increased CY-BOCS scores [21].

The first genome-wide CNV analysis in OCD (1,613 patients) and the largest to date in Tourette Syndrome (TS) (1,086 patients) and 720 ancestry-matched controls was conducted to observe the effects of specific neurodevelopmental CNVs. The analyses were restricted to large (>500 kb an rare (<1% frequency in the Database of Genomic Variants-DGV) CNVs, using Illumina genotyping array. The results showed evidence for an increased burden of pathogenic neurodevelopmental deletions in OCD/TS patients compared with controls. Deletion at 16p13.11 was primarily associated with OCD, however 3 de novo CNVs in this region among 6 cases with OCD/TS point to the possibility of pleiotropic effect of this locus. These deletions at 16p13.11 have been extended to other neurodevelopmental disorders, including developmental delay, seizures, and autism [22].

In light of these findings and given our long-standing interest in the contribution of genetic variation to OCD, we conducted a preliminary study to identify rare CNVs in 16 patients with early-onset OCD and 12 controls, using array-based comparative genomic hybridization (aCGH).

## Materials and Methods

The present study was approved by the Research Ethics Committees of the University of São Paulo School of Medicine, as well as by the Brazilian National Commission of Research Ethics (CONEP, process number: 16756). All participating subjects gave written informed consent.

The study was conducted in the Outpatient Clinic of the Obsessive-Compulsive Spectrum Disorders Program of the Institute of Psychiatry at the General Hospital of the University of São Paulo, School of Medicine.

Patients were referred from primary psychiatric services, or recruited through television, radio, and newspaper announcements. Healthy controls were recruited from university staff and students, hospital staff or by word of mouth.

Patients and healthy controls were assessed at baseline with the Structured Clinical Interview for DSM-IV Axis I Disorders and additional modules for tic and impulse control disorders, the ADHD and separation anxiety sections of the Kiddie Schedule for Affective Disorders and Schizophrenia (K-SADS), the Yale-Brown Obsessive- Compulsive Scale (Y-BOCS), the Dimensional Yale-Brown Obsessive- Compulsive Scale (DY-BOCS), the Yale Global Tic Severity Scale (YGTSS), and the Beck Depression (BDI) and Anxiety (BAI) Inventories [23], [24]. Patients were included if they were aged between 18 and 65 years and had OCD as the primary diagnosis according to DSM-IV criteria. Healthy controls were included if they were aged between 18 and 65 years and did not have OCD, tic or impulse control disorders, ADHD, anxiety disorders or major depression according to the DSM-IV.

Subjects with a primary diagnosis of a psychotic disorder or with any other condition that could impair their understanding of the protocol questions were excluded. Patients with a clinical condition that could hamper the interpretation of the results, such as those whose OCD symptoms began after significant head trauma or were secondary to other neurological disorders and intellectual disability were also excluded.

### Genomic DNA extraction

In those patients from whom it was possible to obtain blood samples, peripheral blood leukocytes were used as a source of DNA, which was extracted using the salting out method [25]. For the eight subjects from whom blood samples could not be drawn, DNA was extracted from saliva samples collected using the Oragene kit manufactured by Genotek according to the protocols available at http://www.dnagenotek.com/.

### Comparative genomic hybridization based on microarray (aCGH)

Array-CGH analysis was performed using an oligonucleotide 44 K whole-genome microarray platform (design 14950, Agilent Technologies, Santa Clara, USA). The platform was composed of 44,290 60-mer oligonucleotide probes for mapped genes or unique DNA sequences with an average spatial resolution of ∼30–35 kb. However, the platform does not cover the pseudoautosomal regions of the X and the Y chromosomes. The genomic DNA of patients and healthy controls was labeled with Cy3-fluorophores, while the reference DNA was labeled with Cy5-fluorophore. These were hybridized to the array in the presence of Cot-1 DNA, a blocking reagent which suppresses the nonspecific hybridization of repetitive sequences.

Test DNA samples were hybridized with gender matched reference DNA, which consisted of a pooled sample of human placenta DNA obtained from ten normal individuals. Purification, hybridization, and washing were carried out according to the manufacturer's instructions. Slides were read by a GenePix 4000B scanner, and the scanned images of the arrays were processed using the Feature Extraction software package (Agilent Technologies, Santa Clara, CA, USA). Fluorescence intensity was measured by the Feature Extraction software, version 9.5, using an Agilent whole human genome 4×44k microarray. Further details on the protocols used are available online (http://www.home.agilent.com/).

These analyses were performed in all patients, healthy controls and additionally, in the parents of one patient who had one rare CNV.

### Selection of high-confidence copy number alterations

Constitutive or germline CNVs were identified using the Genomic Workbench software (Agilent Technologies, Santa Clara, CA, USA) with the statistical algorithm ADM-2, and a 6.7 sensitivity threshold. Poor quality hybridizations (QC>0.3) were discarded. For samples that passed quality control, CNV calling (deletions and duplications) was performed using the Nexus Copy Number 5.0 software, with a quality control threshold of <0.18. CNVs were called in Nexus using the FASST2 segmentation algorithm; with at least three affected probes and default settings (threshold log_2_ ratio of 0.3 or 1.14 for gain or high copy gain, and −0.3 and −1.14 for loss and homozygous loss, respectively), and significance threshold set to 1.0E-6.

Samples that did not meet quality thresholds were excluded from further analysis, as were microarrays with low quality according to visual inspection. Although the initial sample was composed of 20 patients and 20 controls, 12 samples were subsequently excluded for quality reasons (4 patients and 8 controls).

CNVs were considered rare if they showed less than 50% overlap with CNVs predicted with frequency of at least 1% according to the March 2010 update of the Database of Genomic Variants (DGV). The confirmation of rare CNVs and determination of the parent of origin were conducted using 60 K SNP array analysis. Furthermore, rare CNVs were confirmed by quantitative PCR (qPCR).

### Quantitative PCR (qPCR) validation

**Figure 1:**
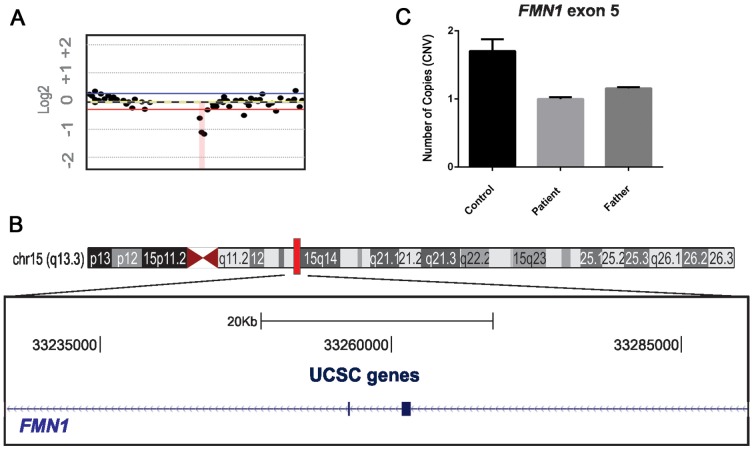
Chromosome 15q 13.3 after CNV and qPCR procedures. **A**. The microdeletion identified in the *FMN1* region of chromosome 15q13.3 using Genomic Workbench software (Agilent Technologies, Santa Clara, CA, USA). **B**. Genome browser image of the region containing the *FMN1* gene. One rare deletion was identified in the male proband (64 kb loss in OCD case 222_3). **C**. Rare CNVs were validated by SYBR Green-based real time PCR, and it was found that the father and son had only one copy of the exon while the gender-matched control had two copies.

Rare CNVs were validated using the SYBR Green system from Applied Biosystems 7500 Real-Time PCR System. Two qPCR primers were designed and mapped against the NCBI reference sequence hg18 to obtain converging evidence for the detected CNVs. Primer sequences were as follows: *FMN1* forward primer: 5′ CAGGTTTGTCTGAAAGTCACC 3′, reverse primer: 5′ CTACTCTTTGTACCTGGGAGGAC -3′. The absolute number of gene copies was normalized using the *GAPDH* and *P2RX7* genes (control genes). Triplicates were analyzed using the comparative 2^−^ΔΔC^t^-cycle threshold method [26]. Values in the 0.8–1.2 range indicated duplication, <0.6 were indicative of deletions, and values>1.4 were considered duplications (Figure 1).

### Statistical Analysis

We assessed the impact of CNVs in cases compared to controls using five measures: the number of CNVs per individual, the estimated CNV size, the percentage of CNVs that overlapped with CNVs in the DGV (overlap with>50% of length), and the percentage of CNVs in two different groups of CNV sizes (CNVs>1 Mb and CNVs>100 kb – 999 kb). *P*-values were calculated using two-tailed Fisher exact test and were shown for each comparison between cases and controls. The level of significance considered was alpha = 0.05 (Table 1).

**Table 1:** Characteristics of all copy number variations (CNVs) in obsessive-compulsive disorder (OCD) patients and controls.

	OCD patients (n = 16)	Controls (n = 12)	*P*-value
	All CNVs	All CNVs	
**Samples with CNVs**	16	12	0.4497
**Number of CNVs**	53	48	0.6188
**Median number of CNVs per genome**	3	2	0.6547
**Median CNV size (kb)**	435.4	389.8	0.1124
**% Gain/Loss**	88.6/11.3	85.4/14.5	0.8083/0.5287
**Overlapping CNVs (%)** 1	58.4	68.7	0.3609
**CNVs>1 Mb (%)**	24.5	20.8	0.5825
**CNVs>100 kb – 999 kb (%)**	67.9	79.1	0.3556

*P*-values were calculated using two-tailed Fisher exact test.

1 Overlapping CNVs = percentage of CNVs that overlap with CNVs in the DGV (overlap with>50% of length).

We compared the proportion of subjects in each group harboring CNVs with sizes between 30 kb–500 kb and>500 kb. This last comparison was conducted to observe whether CNVs involved with neurodevelopment disorder were associated with the OCD group [6]. Pearson's Chi-square (two-tailed) p-values were calculated, using a pre-determined p-value threshold that corrected for 2 primary comparisons using a standard Bonferroni approach (p<0.025) [Table 2].

**Table 2:** Frequency of copy number variants of different sizes in patients with obsessive-compulsive disorder (OCD) and controls.

CNV Size				
Category	Analysis	# OCD (N = 16)	# Controls (N = 12)	*P*-value
All 30–500 kb CNVs	CNVs	53	48	
	Samples	13	11	0.8151
	Proportion	81.2%	91.6%	
All>500 kb CNVs	CNVs	26	26	
	Samples	11	7	0.8644
	Proportion	68.7%	58.3%	

*P*-values were calculated using Pearson's Chi-square test; Bonferroni-adjusted significance of 0.025.

## Results

After completing quality control and case-control matching, 12 samples were excluded (4 cases and 8 controls). Therefore, a total of 16 cases and 12 controls were included in the final analyses. The mean age at symptom onset in the patient group was 9.8 years (SD = 4.03) and the mean Y-BOCS score was 30.4 (SD = 4.93).

### Characteristics of CNVs in patients and controls

The median CNV size in OCD patients was 435.4 kb and the median number of CNVs per individual was 3, with 11.3% of these events consisting of deletions (the remaining were all duplications). A total of 24.5% of CNVs in OCD patients were>1 Mb, while 67.9% of variants were in the 100-999 kb range. Patients and controls did not significantly differ with respect to these characteristics (Table 1).

Overall, no statistically significant differences in CNV size were found between cases and controls, even when a nominal uncorrected p-value of 0.05 was used. Similar results were obtained with regard to between-group differences in the frequency of CNV occurrence (Table 1 and Table 2).

### Rare CNVs analyses

One OCD patient was found to carry a rare CNV consisting of an intragenic ∼64 kb microdeletion at 15q13.3 encompassing part of the *FMN1* gene. Array CGH analyses of the patient's parents were performed to verify whether the CNV was *de novo* or inherited. The microdeletion was found to be inherited from a non-affected father (Figure 1).

## Discussion

In this study an early-onset OCD patient (age of first obsessive-compulsive symptoms when he was 8 years old) was found to carry a rare intragenic ∼64 kb microdeletion at 15q13.3. This microdeletion was present in the father who did not have OCD. The mother also did not have OCD, although his sister also had OCD. Unfortunately the sister did not agree to participate in the study.

This CNV encompasses two exons of the *FMN1* gene, which encodes the protein Formin1. The overexpression of Formin1b produces an increase in the number of primary dendrites in cultured hippocampal neurons and an increase in the number of glutamatergic synaptic inputs [27]. Additionally, it is thought that Formin1 may mediate the effect of the proneural transcription factor Ngn3, which regulates dendritogenesis and synaptogenesis [28].

The genetic findings in OCD has shown dysregulation in genes that are expressed in the brain and are involved in the glutamatergic, serotonergic and dopaminergic pathways playing an important part in the expression of OCD [2].

Recent evidence from a number of studies has highlighted the role of glutamatergic synaptic dysfunctions in the cortico-striatal-thalamo-cortical (CSTC) circuit in the etiology of OCD and related disorders [29]. According to a number of independent reports, one of the strongest candidate genes for OCD is the neuronal glutamate transporter gene *SLC1A1* (Solute Carrier, Family 1, Member 1) [30]–[32]. In fact, this is the only gene whose association with OCD has been replicated in independent samples [4]. However, it is interesting to note that the previously mentioned GWAS of OCD did not find any polymorphisms in the *SLC1A1* gene, although it did associate the disorder with a number of genes involved in glutamatergic processes. These findings corroborate the hypothesis that genes involved in the glutamatergic system may contribute to the pathophysiology of OCD [33], [34].

The OCD Collaborative Genetics Study (OCGS) conducted a linkage analysis on 376 affected families using genetic association methods, and found that the SNPs most strongly associated with OCD were in chromosome 15q. Several of those SNPs are located in or near the *FMN1* gene. One SNP (rs2306277) in particular, located in exon 1 of the *FMN1* gene, was predicted by PolyPhen 2 (a tool which predicts the possible impact of an amino acid substitution on the structure and function of a human protein) to be a missense mutation with probable protein damage. The same study also found SNPs associated with OCD in the same chromosomal region (15q), but in the homeobox genes *MEIS2* and *NANOGP8*, which code for transcription factors or closely related proteins, all of which play an important role in neurodevelopment [35]. Other studies have also reported associations between OCD and the 15q region, although none has identified relevant SNPs in the region of the *FMN1* gene itself [36], [37].

The main CNVs associated with psychiatric disorders have generally been rare variants in genes involved in synaptogenesis, neuronal and axonal mobility, protein degradation via the ubiquitin-proteasome system (ubiquitin signaling), protein complexes and postsynaptic signaling [11], [12], [38]–[48]. Studies have also suggested that the CNVs found in psychiatric probands are often in chromosomal regions associated with microdeletion syndromes, such as loci 16p11.2 and 7q11.23, both associated with Williams Syndrome, locus 15q11-13, associated with Prader-Willi and Angelman Syndromes, and, lastly, locus 22q11.2, which has been associated with DiGeorge syndrome [6].One of the most interesting observations in psychiatric genetic association studies has been the wide variability in the neuropsychiatric phenotype associated with one individual CNV or CNV region [49], [50]. These findings are consistent with the hypothesis linking the known pleiotropic effects of these CNVs with shared abnormalities in early neural development [51].

Interestingly, a microduplication at 15q13.3 (including the first four exons of the CHRNA7 gene) was found in a proband with TS, ADHD and OCD and his mother who had subclinical ADHD. The other brother with TS and OCD (without ADHD) did not have this duplication. The authors suggest that this duplication may have an intermediate penetrance effect and may be involved with ADHD development [52].

Studies have described a broad variety of phenotypes associated with 15q13.3 deletion, including schizophrenia, epilepsy, autism, antisocial behaviors and mental retardation [36], [37], [53]–[56]. The frequency of these deletions in control individuals has been found to be much lower than in cases. Additionally, the types of deletions and duplications may vary between these individuals. The authors suggest that submicroscopic deletions and duplications in this region are associated with milder phenotypes, have less than 100% penetrance, and tend to be inherited in a larger fraction of cases. Environmental factors may also be important in explaining the lack of penetrance [57]. However, none of the studies cited herein have reported the microdeletion found in the present study. This may suggest a pleiotropic effect whereby multiple variants in the same region are associated with different phenotypes [58]. Perhaps the microdeletion found in the present study in conjunction with environmental factors or other genetic variants may contribute for the manifestation of OCD. The overall rate of *de novo* large CNVs (>500 kb) in OCD trio samples was 1.44%, which is intermediate between healthy controls and autism (1.8% multiplex and 3.9% simplex) and schizophrenia (2%–3%). This finding gives some support to the involvement of CNVs in OCD, despite this study was unable to call CNVs smaller than 500 kb due to technical limitations. There is still the possibility that smaller CNVs may be related to OCD [22].

Several limitations of the present study are worth mentioning. Firstly, the sample size was small, particularly for the purposes of the detection and analysis of rare CNVs. Although this association may have happened by chance alone, other possibilities such as incomplete penetration can be taken into account. Examining the affected sister could help understand this issue.

Furthermore, even though the 44 K platform allows for the observation of large and rare CNVs; the probe spacing may affect the accuracy of CNV length measurements. Future analyses should be conducted using larger sample sizes and a better platform resolution. However, regardless of these limitations, the present findings give further support to the hypothesis that many variations in genes that function in a brain network can be related with OCD, instead of single genes that simply cumulatively add risk.
